# An Explainable Multimodal Framework for Breast Ultrasound Report Generation Using Vision-Language Transformers

**DOI:** 10.3390/jimaging12080338

**Published:** 2026-07-27

**Authors:** Prashanth Gowda Attahalli Shivakumar, Azhar Mahmood, Shaheen Khatoon

**Affiliations:** Department of Computer Science and Digital Technologies, School of Architecture, Computing, and Engineering, University of East London, London E16 2RD, UK; u2828998@uel.ac.uk (P.G.A.S.); s.khatoon@uel.ac.uk (S.K.)

**Keywords:** automatic report generation, swin transformer, ClinicalBERT, GPT-2, breast ultrasound, multimodal learning

## Abstract

Breast cancer remains one of the leading causes of cancer-related mortality among women worldwide, where early and accurate diagnosis is critical for effective treatment. Although recent advances in deep learning have enabled automated radiology report generation from breast ultrasound images, most existing approaches function as black-box systems, limiting clinical trust and interpretability. This study proposes a trustworthy and explainable framework for automated breast ultrasound report generation that combines Vision-Language Modelling (VLM) with multi-level Explainable Artificial Intelligence (XAI). The proposed architecture integrates a Swin Transformer for visual feature extraction, BioBERT/ClinicalBERT for clinical text representation, and a GPT-2-based decoder for report generation through a dual cross-attention fusion mechanism. The framework is evaluated on benchmark breast ultrasound datasets paired with expert-annotated radiology reports using standard natural language generation metrics, including BLEU, ROUGE-L, METEOR, and CIDEr. Experimental results demonstrate that the multimodal architecture significantly improves report quality, clinical consistency, and semantic accuracy compared with conventional image-only and single-modal baselines. To address transparency and trustworthiness, the framework provides dual-level explanations through Grad-CAM visual heatmaps and LIME/SHAP-based token attribution analysis, enabling clinicians to understand both image regions and textual features influencing generated reports. Qualitative assessment further indicates strong alignment between model explanations and radiologist-identified diagnostic findings.

## 1. Introduction

Globally, breast cancer remains one of the most frequently diagnosed malignancies and a leading contributor to cancer-related mortality among women [1], making early and precise diagnosis critical to improving clinical outcomes. Breast ultrasound occupies a central role in screening and diagnostic protocols, particularly for patients with dense breast tissue where mammographic sensitivity is substantially reduced [2]. Ultrasound offers several practical advantages as an imaging modality: it involves no ionizing radiation, is comparatively low-cost, provides real-time imaging, and is widely accessible, including in resource-constrained and point-of-care settings [3]. Supplemental ultrasound screening has been shown to improve cancer detection in women with dense breasts beyond mammography alone [4]. However, its diagnostic effectiveness remains highly operator-dependent, relying on radiologist expertise in image acquisition and interpretation, which introduces inter-observer variability [5].

Radiological assessment requires careful evaluation of lesion morphology, tissue architecture, margin definition, and echogenic patterns prior to composing a structured diagnostic report. This interpretative workflow is inherently labour-intensive and may introduce variability between observers. As a result, automated reporting systems [4,6,7,8] have emerged as a promising solution to enhance consistency, streamline clinical workload, and support evidence-based decision-making.

In recent years, the field of automatic report generation within medical imaging has attracted considerable and growing scholarly attention, a trend driven in large part by the remarkable advances witnessed in deep learning methodologies and the emergence of Transformer-based architectures as the dominant paradigm for sequence modelling tasks [5]. The earliest contributions to this domain drew heavily upon encoder–decoder configurations grounded in convolutional and recurrent neural network frameworks, adapting concepts and architectural principles originally developed within the broader image captioning literature for application to the specific demands of medical image interpretation [9,10]. While these foundational models made a significant intellectual contribution by demonstrating the fundamental feasibility of establishing learnable mappings between visual inputs and textual outputs in a clinical context, they were not without substantial limitations. In particular, such architectures frequently proved inadequate in their capacity to represent extended contextual dependencies spanning the full length of a radiology report, and they similarly struggled to faithfully capture the nuanced and domain-specific clinical semantics that distinguish authentic radiological documentation from general-purpose descriptive text, thereby exposing clear boundaries to what early encoder–decoder approaches could achieve in this domain.

Subsequent research addressed these limitations through attention-based mechanisms that guide models toward diagnostically relevant image regions [11,12]. More recent Transformer-driven approaches further strengthened contextual modelling by capturing global relationships across visual and textual modalities, thereby improving linguistic coherence, diagnostic relevance, and overall report quality [13].

Despite substantial progress in automatic report generation, breast ultrasound remains comparatively underexplored, with existing studies often relying on small benchmark datasets of approximately 256 cases [14]. Modality itself introduces distinctive challenges. Ultrasound images are characterized by low signal-to-noise ratios, heterogeneous lesion presentation, and operator-dependent variability, all of which complicate both visual interpretation and the generation of precise, clinically appropriate diagnostic language.

Recent research [6,15,16] has begun to address these challenges through multimodal Transformer-based strategies tailored to breast ultrasound reporting. These approaches combine vision encoders with domain-adapted language models to bridge image-derived features and biomedical semantics within a unified framework. Integrating a Vision Transformer with pre-trained language representations such as BERT variants and autoregressive decoders has demonstrated improvements in clinical specificity and linguistic fluency. Cross-modal attention mechanisms further enhance alignment between visual patterns and textual descriptions, supporting more coherent and diagnostically meaningful report generation.

Although these studies provide a solid baseline for automated breast ultrasound report generation, several limitations persist. (i) Relying on a standard Vision Transformer without hierarchical feature modelling may constrain the model’s ability to capture fine-grained lesion morphology and multi-scale spatial patterns. (ii) The dependence on global image embeddings can reduce lesion-level grounding, potentially resulting in more template-driven outputs. (iii) The small dataset size limits the model’s generalization across diverse ultrasound acquisition conditions. Finally, despite improvements in linguistic coherence, the approaches do not explicitly incorporate structured diagnostic reasoning into the report generation process.

To address these challenges, this study proposes a lesion-aware hierarchical Transformer approach that enhances spatial feature extraction, strengthens multimodal alignment, and introduces auxiliary supervision to improve visual grounding under small-data constraints. By refining the visual backbone and improving multimodal conditioning strategies, this research aims to enhance diagnostic reliability, generalization performance, and interpretability in automated breast ultrasound report generation. The main contributions of this study are as follows:

1. Lesion-aware multimodal framework: We propose an end-to-end Transformer framework integrating a hierarchical Swin visual encoder with mask-guided attention, a domain-adapted ClinicalBERT text encoder, and a GPT-2 decoder, unified through a dual cross-attention fusion mechanism. Unlike prior work relying on flat Vision Transformers and global image embeddings, this design explicitly captures fine-grained lesion morphology and strengthens lesion-level visual grounding.
2. Improved clinical performance: Through a component-level ablation study, we demonstrate that each architectural element mask-guided attention, dual cross-attention, and domain-adapted text encoding contributes measurable gains across BLEU, ROUGE-L, METEOR, and CIDEr, establishing the necessity of each design choice rather than assuming it.
3. Clinically coherent outputs: The framework produces structured reports aligned with expert references, and its decision process is validated through a multi-method explainability analysis (Grad-CAM, Grad-CAM++, attention rollout, per-head cross-attention, LIME, and token-level attribution) with quantitative faithfulness evaluation, addressing the transparency requirements essential for clinical trust.
4. Model interpretability: Validates transparency using Grad-CAM, LIME, and attention visualization, showing focus on diagnostically relevant regions and terms.

## 2. Related Work

Recent progress in radiology report generation has been largely shaped by encoder–decoder paradigms. Many of these methods trace their roots to image captioning research, where the central task involves producing a structured and meaningful textual description from visual input [10,11,12,17]. In most implementations, a convolutional neural network (CNN) functions as the visual encoder, extracting salient and discriminative features from medical images. These representations are then passed to a language generation module, typically a recurrent neural network (RNN) or a Transformer-based decoder, which constructs the associated radiological report by modelling sequential dependencies within the text.

While early systems relied on CNN–RNN pipelines, contemporary research increasingly leverages transfer learning, Transformer architectures, and large language models to improve semantic coherence and clinical relevance. Jin et al. [18] introduced a multi-grained abnormality prediction framework that aligns pre-trained visual features with anatomical and disease-related concepts, reporting gains in both language quality and clinical metrics. However, its reliance on predefined medical concept taxonomies constrains adaptability to atypical cases or institutions with heterogeneous reporting styles.

In the domain of breast cancer diagnostics, Nguyen and Theodorakopoulos [19] proposed a text-focused encoder–decoder system with a dedicated classification module for automating BI-RADS categorization, demonstrating measurable gains in structured score prediction on Dutch radiology reports. However, the approach operates exclusively on textual data; the absence of visual feature integration limits its applicability to multimodal ultrasound reporting, where lesion morphology, echogenicity, and boundary characteristics are central to diagnostic reasoning.

Himel and Chowdhury [20] proposed an encoder–decoder framework for automated segmentation and classification of breast lesions in ultrasound, using transfer learning with pre-trained backbones (ResNet50V2, NASNetLarge, EfficientNetB7) and a dual-stage feature fusion strategy to improve discrimination between visually similar benign and malignant lesions. Evaluated on the BUSI and Thammasat datasets, it achieved 70.82% IoU and 80.56% Dice. Although not focused on report generation, its hierarchical feature extraction and multi-scale representation learning offer relevant insights for the lesion-level grounding required in multimodal breast ultrasound reporting.

Tao and Ma [8] proposed a memory-enhanced framework that incorporates semantic retrieval into multimodal learning, combining CNN-based visual features with a cross-modal memory module storing disease-related representations from radiology corpora. The memory mechanism supplies relevant clinical knowledge during generation, and evaluation on MIMIC-CXR and IU X-Ray showed consistent improvements across standard metrics.

Chaudhury and Sau [21] integrated Vision Transformers with an LSTM-based classifier for breast cancer image classification, treating image patches as sequential inputs to capture global spatial context. The framework achieved 99.2% accuracy on a histopathology dataset and generalized effectively to the BUSI ultrasound dataset, highlighting the representational strength of Transformer-based vision encoders over conventional convolutional architectures for breast image analysis.

Singh and Singh [7] presented ChestX-Transcribe, a Transformer-based multimodal system for chest X-ray report generation that combines a Swin Transformer for hierarchical visual encoding with a lightweight language model. Evaluated on the IU X-Ray benchmark, it outperformed prior methods in clinical consistency and contextual relevance. Although designed for chest radiography, its multi-scale visual encoding and Transformer-based cross-modal alignment are transferable to breast ultrasound, where precise lesion representation and image–text correspondence are critical.

More broadly, the emergence of large language models has significantly influenced recent radiology report generation research. Wang and Liu [16] proposed R2GenGPT, which couples Transformer-based visual encoding with a large-scale autoregressive language model to enhance narrative fluency and semantic structure. Experiments on established chest X-ray datasets showed gains across standard language generation benchmarks following extensive computational training. However, performance improvements were not uniformly observed across all evaluation criteria, and certain competing models demonstrated stronger results on consensus-oriented metrics. Additionally, reliance on annotated radiology corpora and the absence of ensemble learning strategies may limit robustness and adaptability in heterogeneous clinical settings.

Khatoon and Mahmood [6] presented a Transformer-based multimodal framework tailored to breast ultrasound report generation, aiming to align image features with biomedical language representations. Their approach combines vision-based encoding with domain-adapted language modelling, enabling cross-modal interaction during structured diagnostic narration. Rather than relying solely on visual embeddings, the system integrates pretrained clinical language models to strengthen semantic grounding and improve textual coherence. Architecture employs attention-driven mechanisms to associate image-derived representations with textual embeddings during autoregressive generation. Experimental evaluation on the BrEaST dataset demonstrated measurable improvements in commonly used report generation benchmarks, particularly when domain-specific language modelling was incorporated. However, training the visual encoder from scratch under computational constraints may restrict its ability to capture hierarchical and multi-scale lesion characteristics. These limitations highlight the need for larger datasets, pre-trained hierarchical vision encoders, and stronger lesion-level alignment strategies to enhance generalization and diagnostic reliability.

Tsai and Chu [22] developed an automated gallbladder ultrasound reporting framework using Faster R-CNN for lesion detection and spatial localization, combined with an LSTM-based decoder to generate structured diagnostic reports from visual features. The pipeline maps detected pathological regions into coherent clinical narratives. To improve reliability, a weight selection mechanism was introduced to emphasize diagnostically informative features. The study shows that relying solely on image features is insufficient for robust reporting, and highlights the importance of incorporating domain-specific clinical knowledge to enhance accuracy and contextual understanding in report generation.

Ucan and Kaya [23] introduced Model-SEY is a hybrid encoder–decoder framework for automated Turkish chest X-ray report generation, combining a Swin Transformer visual encoder with a Turkish-adapted GPT model (cosmosGPT) as the decoder. The Swin Transformer extracts multi-scale radiographic features via shifted-window attention, while cosmosGPT generates clinically structured Turkish reports aligned with radiological terminology. The model was trained on 5998 image–report pairs from Elazığ Fethi Sekin City Hospital and the Indiana University Chest X-Ray dataset, with English reports translated into Turkish and clinically validated. It achieved strong performance (BLEU-1: 0.6412, BLEU-4: 0.3716, ROUGE: 0.2240), and expert radiologists confirmed the clinical quality of outputs. However, evaluation was limited to controlled settings and Transformer-only architectures, leaving questions about comparison with CNN-based methods and real-world clinical scalability.

Raminedi and Shridevi [24] presented a multimodal Transformer architecture integrating Vision Transformer variants (ViT, BEiT, DEiT) as visual encoders with a GPT-2 decoder, using cross-attention to align visual and textual representations. Evaluated on the IU X-Ray dataset, it demonstrated strong performance in both image interpretation and report generation. However, its dependence on high-quality imaging data and substantial computational resources may limit scalability and practical deployment in resource-constrained clinical settings.

## 3. Materials and Methods

This study proposes an explainable multimodal AI framework for automated radiology report generation from breast ultrasound images. The framework is designed to imitate the diagnostic workflow of radiologists, who do not rely solely on image appearance but combine visual findings with structured clinical descriptors before formulating a diagnostic interpretation. Accordingly, the proposed model integrates breast ultrasound images, tumour segmentation masks, structured clinical attributes, and reference radiology reports into a unified multimodal report-generation pipeline.

**Figure 1:**
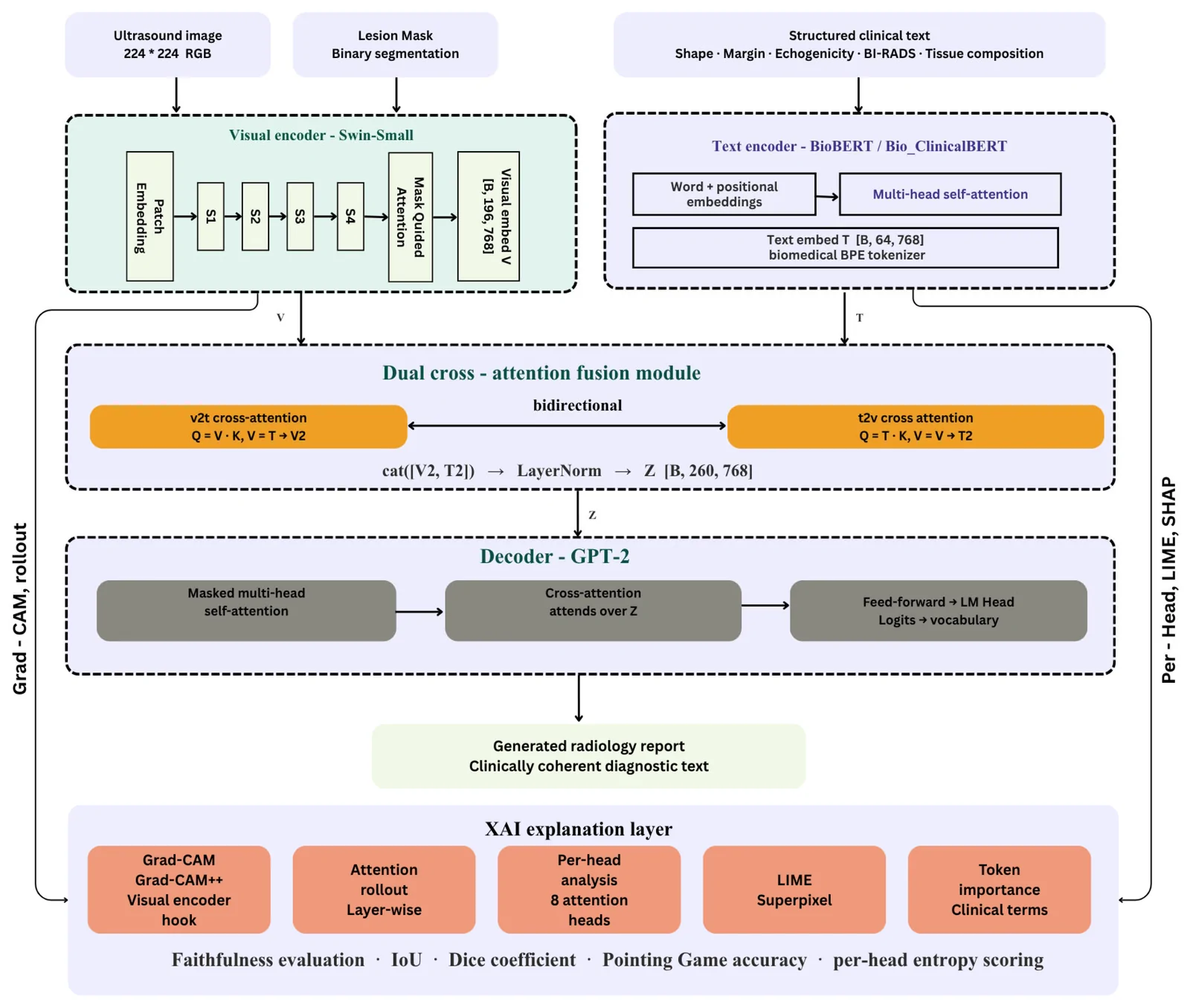
Explainable multimodal framework for breast ultrasound report generation.

The overall architecture is shown in Figure 1, which consists of five main components: a visual encoder, a text encoder, a dual cross-attention fusion module, a transformer-based report decoder, and an explainability and model interpretation module. First, the breast ultrasound image is processed by a Swin Transformer-based visual encoder to extract hierarchical image features. Tumour segmentation masks are then used to guide attention toward clinically important lesion regions. In parallel, structured clinical attributes, including tissue composition, lesion shape, margin, echogenicity, and BI-RADS category, are converted into natural language sequences and encoded using a biomedical language model. The visual and textual embeddings are then aligned through a bidirectional cross-attention mechanism, allowing the model to learn relationships between image regions and clinical descriptors. Finally, the fused multimodal representation is passed to a GPT-2-based transformer decoder, which generates a coherent radiology report conditioned on both imaging and clinical information.

The proposed framework differs from conventional image-captioning systems by explicitly incorporating multimodal clinical context and explainability mechanisms. This is important in medical imaging, where model predictions must be clinically interpretable, transparent, and traceable to relevant findings. Transformer-based architectures are particularly suitable for this task because attention mechanisms allow the model to capture long-range dependencies within images, text, and multimodal feature representations [25,26].

### 3.1. Visual Encoder

The visual encoder serves as the image-understanding component of the proposed framework. It extracts diagnostically meaningful representations from raw breast ultrasound images. In this study, the Swin Transformer is adopted as the visual backbone because of its hierarchical structure and shifted-window self-attention mechanism [25]. Unlike conventional convolutional neural networks, which process images through fixed local receptive fields, the Swin Transformer divides an image into non-overlapping windows and applies self-attention within each window. By shifting the window partitions across successive layers, the model enables information exchange between neighbouring regions while maintaining computational efficiency.

Internally, the input image *I* is first divided into small image patches, which are projected into token embedding’s. These tokens are then processed through multiple transformer stages. Each stage applies window-based multi-head self-attention, followed by feed-forward neural layers and normalization operations. Patch merging is used between stages to reduce spatial resolution while increasing feature dimensionality. This hierarchical design allows the model to capture both fine local details and broader structural patterns, such as lesion shape, boundary regularity, and surrounding tissue context.

(1) $$X_{0}=PatchEmbed\left(I\right)+E_{p}$$

where $X_{0}$ is the initial patch-token representation and $E_{p}$ is the positional embedding. Within each Swin Transformer block, window-based self-attention is applied as:

(2) $$Attention\left(Q,K,V\right)=softmax\left(\frac{QK^{T}}{\sqrt{d}}+B\right)V$$

where Q,K and V are query, key, and value matrices, d is the feature dimension, and B is the relative position bias. This equation represents how the Swin Transformer models relationships between image patches within local windows while preserving spatial structure. Each layer applies residual connections and layer normalization as follows:

(3) $${\tilde{X}}^{\left(l\right)}=W-MSA\left({LN(X}^{\left(l-1\right)})\right)+X^{\left(l-1\right)}$$

(4) $$X=MLP\left({\tilde{X}}^{\left(l\right)}\right)+{\tilde{X}}^{\left(l\right)}$$

where *W-MSA* denotes window-based multi-head self-attention, *LN* is layer normalization, and *MLP* is the feed-forward network.

To improve the clinical focus of the visual encoder, tumour segmentation masks are incorporated into the image-processing pipeline. The purpose of this component is to guide the model toward lesion-specific regions while retaining useful contextual information from surrounding tissue. This is important because radiology reports usually describe both the lesion itself and its relationship to adjacent anatomical structures.

After the Swin Transformer generates image feature maps, the tumour mask is resized or projected to match the spatial dimensions of the extracted feature maps as follow:

(5) $$\tilde{M}=Resize\;(M)$$

The mask is then used to modulate the feature representation by increasing the contribution of pixels or tokens corresponding to the lesion region. This produces a lesion-aware feature map in which diagnostically important areas receive greater attention. However, the surrounding tissue is not completely removed, since contextual features may also contribute to interpretation, particularly in assessing lesion margins, posterior acoustic features, and tissue composition. The extracted visual features $f_{v}$ are then modulated using the resized mask:

(6) $$Z_{v}=\;f_{v}\;ʘ\;(1+\;⅄\tilde{M)}\;$$

where $Z_{v}$ is the lesion-aware visual representation, ʘ denotes element-wise multiplication, and ⅄ controls the strength of lesion emphasis. This equation mathematically supports the description that tumour regions are emphasized while surrounding tissue context is retained. This mask-guided mechanism helps reduce the risk that the model will focus on irrelevant background structures or artefacts. It also improves interpretability, as the model’s visual reasoning becomes more directly associated with known tumour locations.

### 3.2. Text Encoder

The text encoder processes structured clinical information associated with each ultrasound case. These structured attributes include tissue composition, lesion shape, margin characteristics, echogenicity, and BI-RADS category. Since transformer-based language models operate on textual sequences, the structured attributes are first converted into natural language statements. For example, a case may be represented as: “*The lesion has an irregular shape, indistinct margin, hypoechoic echogenicity, and BI-RADS category 4.*”

BioBERT is used as the biomedical text encoder because it is pre-trained on large-scale biomedical corpora and is able to represent clinical and biomedical terminology more effectively than general-domain language models [27]. BioBERT builds on the original BERT architecture, which uses bidirectional self-attention to learn contextual relationships between words [26]. ClinicalBERT further demonstrates the value of domain-specific pre-training for clinical language understanding, particularly when dealing with medical documentation and clinical note structures [28].

The structured clinical attributes are first tokenized and converted into embeddings. These embeddings pass through multiple transformer layers, where self-attention enables each token to attend to all other tokens in the sequence. As a result, the model does not encode each clinical attribute in isolation; rather, it learns relationships among attributes. For example, the combination of irregular shape, spiculated margin, and suspicious BI-RADS category may be represented as a clinically meaningful pattern. The final output of the text encoder is a contextualized embedding that captures the semantic meaning of the structured clinical information as follow:

(7) S= g(A)

(8) $$Z_{t}=E_{t}(S)$$

(9) $$H_{t}=TransformerBlock(H_{l-1})$$

where S represents the textual description of attributes such as tissue composition, lesion shape, margin, echogenicity, and BI-RADS category, $Z_{t}$ is the contextual embedding of generated textual sequence using BioBERT or ClinicalBERT.

### 3.3. Dual Cross-Attention Mechanism

The dual cross-attention module is the central fusion component of the proposed framework. Its purpose is to align the visual features extracted from ultrasound images with the textual features derived from structured clinical attributes. This alignment is essential because clinically accurate report generation requires the model to connect image regions with relevant diagnostic descriptors.

The module performs two complementary attention operations. In the first direction, visual tokens act as queries, while textual embeddings act as keys and values calculated as:

(10) $$C_{v\leftarrow t}=Softmax\left(\frac{Q_{v}K_{t}^{T}}{\sqrt{d}}\right)v_{t}$$

This allows image features to attend to relevant clinical descriptors. For example, visual tokens corresponding to an irregular lesion boundary may attend strongly to textual descriptors related to lesion margin or shape. In the second direction, textual embeddings act as queries, while visual tokens act as keys and values. This allows clinical descriptors to attend back to relevant image regions calculated as:

(11) $$C_{t\leftarrow v}=Softmax\left(\frac{Q_{t}K_{v}^{T}}{\sqrt{d}}\right)v_{v}$$

The outputs from these two attentions are then concatenated to form a unified multimodal representation. This representation contains information from both modalities and captures their interactions. A self-attention layer is subsequently applied to refine the fused representation by modelling internal dependencies within the combined visual-textual feature space calculated as:

(12) $$C=\;\left[C_{t\leftarrow v};\;C_{v\leftarrow t}\right]$$

(13) $$Z_{m}=SelfAttention\;(C)$$

where $Z_{m}$ is the final multimodal representation. This process allows the model to generate reports that are not only linguistically coherent but also grounded in both image evidence and clinical context.

### 3.4. Transformer-Based Decoder

The decoder is responsible for generating the final radiology report. In this framework, GPT-2 is used as the generative backbone because it is an autoregressive transformer language model capable of producing coherent and contextually consistent text [29]. GPT-2 generates text sequentially, predicting each new token based on the tokens that have already been generated.

In the proposed framework, GPT-2 is modified to incorporate cross-attention layers. These layers allow the decoder to condition its output on the fused multimodal representation produced by the dual cross-attention module. During each decoding step, the model attends to the previously generated report tokens through masked self-attention. The mask ensures that the decoder only uses previous tokens and does not access future tokens during training. The decoder then uses cross-attention to retrieve relevant information from the fused image-text representation as follow:

(14) $$h_{t}=D_{o\;(y<t),\;}\;Z_{m}$$

(15) $$P(y_{t}|y<t,\;Z_{m})=Softmax(W_{o}h_{t})\;$$

where $h_{t}$ is the decoder hidden state and $W_{o}$ is the output projection matrix. These equations support the description that report generation is conditioned on both previously generated text and multimodal image-clinical evidence. The final report is generated as:

(16) $$\hat{Y}={arg\;max}_{Y\;}\;\prod_{t=1}^{T}P(y_{t}|y<t,\;Z_{m})$$

This design enables the generated report to align image and associated clinical attributes. For example, when generating a phrase related to lesion margin, the decoder can attend to both the visual features representing lesion boundaries and the clinical text embedding describing the margin. This helps improve the clinical consistency and relevance of the generated report.

### 3.5. Explainability and Model Interpretation

Explainability is incorporated into the framework to improve transparency and clinical trust. In medical AI systems, it is not sufficient for a model to generate a plausible report; the system should also provide evidence indicating which image regions and clinical attributes contributed to the generated output. XAI is particularly important in healthcare because clinicians must be able to assess whether the model’s reasoning is consistent with established diagnostic principles [30]. In this work, three explainability methods were explored: Grad-CAM, LIME, and SHAP.

Grad-CAM is used to interpret the visual encoder because it highlights image regions that contribute most strongly to a model output using gradient-based activation maps [31]. This is suitable for breast ultrasound report generation because the heatmaps can be compared with tumour segmentation masks to verify whether the model focuses on clinically relevant regions such as the lesion area and boundary.

LIME is used for local case-level interpretation because it explains individual predictions by perturbing the input and fitting an interpretable surrogate model around the specific case [32]. This makes it useful for assessing how changes in ultrasound regions or clinical attributes, such as lesion shape, margin, echogenicity, and BI-RADS category, influence a generated report.

SHAP is used for feature contribution analysis because it provides additive feature-attribution values based on Shapley values from cooperative game theory [33]. This is appropriate for evaluating the relative influence of structured clinical variables and multimodal features on generated diagnostic content. In addition, attention and cross-attention maps were analyzed to link generated report phrases with relevant image regions and clinical descriptors. Together, Grad-CAM, LIME, SHAP, mask-guided grounding, and attention analysis supported visual localization, feature attribution, error analysis, and clinical validation.

The explainability components serve three concrete functions within a clinical review workflow rather than acting purely as post hoc visualizations. First, the Grad-CAM and Grad-CAM++ heatmaps function as a visual audit of the model’s reasoning a reviewing radiologist can verify whether the predictive evidence originates from the lesion itself or from irrelevant background tissue, speckle artefacts, or acquisition boundaries. A report generated from spatially implausible evidence can therefore be identified and rejected even when its language appears fluent, providing a practical safeguard against hallucinated findings. Second, the per-head cross-attention maps expose which clinical descriptor is grounded in which anatomical region, enabling targeted review; if a report asserts posterior acoustic enhancement, the corresponding attention head can be inspected to confirm that the model attended to the tissue region inferior to the lesion where such phenomena manifest. Third, the token-level importance scores provide a traceable rationale for the diagnostic conclusion by revealing which structured clinical attributes most influenced generation. Collectively, these mechanisms address the transparency requirements that underpin clinical trust in AI-assisted diagnostic tools.

SHAP was implemented using a PartitionExplainer with a blur-based image masker at a target resolution of 128 × 128 pixels. However, obtaining stable Shapley estimates proved computationally prohibitive for the proposed architecture, as each perturbation requires a complete multimodal forward pass through the visual encoder, text encoder, fusion module, and autoregressive decoder. Empirical measurement yielded evaluation times exceeding five minutes for only two explainer iterations far short of the sample count required for convergence. Given that LIME provides comparable region-level attributions at substantially lower computational cost, and that Grad-CAM++ and attention-based analyses supply complementary spatial and cross-modal perspectives, SHAP results are not reported in the final evaluation. This reflects the computational characteristics of Shapley-based attribution applied to autoregressive multimodal generation rather than a limitation of the method itself.

## 4. Experimental Results

### 4.1. Dataset Description

This study uses the Breast-Lesions-USG (BrEaST) dataset [14], a benchmark for breast ultrasound analysis comprising 256 clinical images collected in Poland between 2019 and 2022, each paired with radiological reports. All cases were expert-annotated by a specialist radiologist, ensuring high-quality labels and diagnostic reliability.

The dataset includes 154 benign, 98 malignant, and 4 normal cases. Lesion regions were manually delineated using freehand segmentation, producing 266 masks in total to account for multiple lesions in some scans. Lesions are also categorized using the BI-RADS classification system, ensuring standardized clinical interpretation. All cases include verified clinical outcomes from follow-up or biopsy, making the dataset a reliable resource for developing and evaluating automated breast lesion analysis systems. The four normal cases were excluded from this study, as they contain no lesion segmentation masks and are therefore incompatible with the mask-guided attention mechanism employed by the visual encode all experiments were conducted on the remaining 252 lesion-bearing cases.

The BrEaST dataset has been widely used for breast ultrasound tasks such as image classification, lesion detection, and diagnostic prediction [6]. However, most prior studies focus on image-level analysis. To the best of our knowledge, very few have explored its use for automated report generation or multimodal image and text learning, making this a relatively underexplored area.

### 4.2. Data Partitioning and Evaluation Protocol

The 252 lesion-bearing cases were partitioned into training, validation, and test subsets comprising 176, 38, and 38 cases respectively, corresponding to an approximate 70/15/15 split. The partition was stratified by lesion classification to preserve the benign-to-malignant ratio across all three subsets and was generated using a fixed random seed (random_state = 42) to ensure full reproducibility of the reported results. The resulting class distribution is summarized in Table 1.

All quantitative metrics reported in this study were computed exclusively on the held-out test set, which was not exposed to the model at any stage of training or model selection. The validation set was used solely to monitor generalization performance and to determine the early-stopping point based on validation loss. As no test-set information influenced training or model selection, the reported results constitute an unbiased estimate of model performance on unseen data. The same partition was applied consistently across all experimental configurations, including the encoder comparison and the component-level ablation study, ensuring that performance differences are attributable to architectural factors rather than to variation in data splits.

### 4.3. Data Preprocessing

All ultrasound images were resized to a uniform resolution of 224 × 224 pixels to satisfy the input dimensionality requirements of the Swin-Small visual encoder. Following resizing, pixel intensity values were normalized across the dataset to ensure a consistent and stable intensity distribution, thereby reducing the risk of the model developing sensitivity to variations in image brightness or contrast that are incidental to the underlying clinical content. To further promote generalization and mitigate the risk of overfitting given the relatively modest scale of the dataset, a set of basic data augmentation operations were applied during training, encompassing horizontal flipping and slight rotational transformations that introduce representational diversity while preserving the anatomical validity of the ultrasound content.

The binary lesion segmentation masks provided within the dataset were resized to correspond with the spatial resolution of the feature maps produced by the Swin-Small encoder, specifically a resolution of 7 × 7, ensuring precise spatial alignment between the mask regions and the visual feature representations to which they are applied. During the feature extraction phase, these resized masks were incorporated as a guiding signal within the attention mechanism, directing the model’s representational focus toward the clinically relevant lesion regions and discouraging undue attention to diagnostically uninformative background areas.

The structured clinical attributes and radiology report texts contained within the dataset were subjected to a standardized preprocessing pipeline prior to model input. This involved the conversion of all textual content to lowercase and the systematic removal of extraneous special characters that would otherwise introduce noise into the tokenization process. The cleaned text sequences were subsequently tokenized using modality appropriate tokenizers, with the ClinicalBERT tokenizer applied to the structured clinical attribute inputs and the GPT-2 tokenizer employed for the radiology report sequences designated as generation targets. To ensure uniformity of input length across all samples and to prevent the model from attending to padding tokens during training, padding was applied consistently across all sequences and accompanied by the generation of appropriate attention masks that explicitly exclude padded positions from the model’s attention computations.

### 4.4. Evaluation Metrics

The performance of the proposed Transformer based architecture is assessed through a suite of established natural language generation metrics, each of which has been widely validated within the broader text generation literature and, more specifically, within the domain of automated medical report evaluation. The metrics employed in this study comprise BLEU [34], ROUGE-L [35], METEOR [36], and CIDEr [37], collectively providing a multidimensional perspective on the linguistic quality and fidelity of the generated outputs relative to their corresponding reference reports.

Among the evaluation metrics used for automated text quality assessment, BLEU (Bilingual Evaluation Understudy) is one of the most widely adopted. Originally developed for machine translation, it has since been applied to various text generation tasks, including automated medical report generation. BLEU measures the degree of lexical overlap between a generated report and one or more reference texts by computing n-gram precision across different sequence lengths, thereby providing a measure of surface-level textual similarity [34].

(17) $$BLEU=BP\times exp\left(\frac{1}{N}\sum_{n=1}^{N}log(Pn)\right)$$

where (*BP*) is the brevity penalty that discourages excessively short outputs, (*N*) is the maximum n-gram order considered, and (*P_n*) represents the precision of matching n-grams of order (*n*). Higher BLEU scores indicate greater lexical similarity between the generated and reference reports.

ROUGE-L [35] evaluates the similarity between a generated report and a reference text based on the Longest Common Subsequence (LCS), allowing matched words to appear in a non-consecutive order. Unlike BLEU, which focuses primarily on n-gram precision, ROUGE-L captures sequence-level similarity and emphasizes content coverage, making it well suited for medical report generation where completeness is important.

(18) $$ROUGE-L=\frac{LCS(C,R)}{R}$$

where *LCS*(*C*,*R*) represents the length of the longest common subsequence between the candidate *(C)* and reference (*R*) texts, and |*R*| denotes the length of the reference sequence.

METEOR [36] evaluates the similarity between generated and reference texts using a more linguistically informed approach than BLEU. In addition to exact word matches, it considers stemmed words and synonyms, enabling it to capture semantic similarities despite variations in wording. The metric combines unigram precision and recall, with greater emphasis on recall, and applies a fragmentation penalty to discourage disjointed or poorly ordered text. As a result, METEOR provides a more comprehensive assessment of content coverage and textual coherence, making it particularly suitable for medical report generation.

(19) $$METEOR=F_{mean}\times \left(1-P_{penalty}\right);P_{penalty}=\gamma {\left(\frac{ch}{m}\right)}^{\theta }$$

where *ch* denotes the total number of matched contiguous chunks identified between the generated output and the reference text, m represents the cumulative count of matched unigrams across the aligned sequence pair, and *γ* and *θ* are learnable parameters whose values are adjusted during configuration to govern the degree and sensitivity of the fragmentation penalty applied to the overall score.

CIDEr [37] measures similarity between a generated report and its reference using cosine similarity over TF–IDF weighted n-gram representations. It assigns higher weights to n-grams that are consistent across multiple reference annotations, reflecting their consensus-based importance, while down-weighting commonly occurring phrases that are less informative. This emphasis on distinctive, clinically relevant terms makes CIDEr particularly suitable for evaluating medical report generation. The metric is formally defined as follows:

(20) $$CIDEr=\frac{1}{N}\sum_{n=1}^{N}\frac{{\vec{g}}_{n}\times {\vec{r}}_{n}}{\left|{\vec{g}}_{n}\right||{\vec{r}}_{n}|}$$

where ${\vec{g}}_{n}$ and ${\vec{r}}_{n}$ denote the TF-IDF weighted n-gram vectors corresponding to the generated report and the reference report respectively, and *N* represents the maximum n-gram order considered in the evaluation. The CIDEr score is subsequently computed by calculating the cosine similarity between these weighted vector representations at each individual n-gram level and averaging the resulting similarity values across all n-gram orders, thereby producing a single composite score that reflects the consensus between the generated output and the reference annotations.

Collectively, the evaluation metrics provide a comprehensive assessment of automatically generated ultrasound reports from complementary perspectives. BLEU measures surface-level lexical overlap, ROUGE-L captures structural similarity via sequence alignment, METEOR accounts for semantic variation through synonym and stem matching, and CIDEr emphasizes clinically relevant, consensus-based content. Together, these metrics ensure a balanced evaluation of linguistic quality, coherence, semantic fidelity, and clinical relevance in automated breast ultrasound report generation.

## 5. Result and Analysis

The proposed model is trained under a supervised learning paradigm using cross-entropy loss, computed between decoder-generated token sequences and ground truth report sequences. This provides a token-level training signal that encourages the model to learn an accurate vocabulary distribution at each generation step. Parameters are optimized using the AdamW optimizer with a learning rate of 2 × 10^−5^ and a batch size of 16, balancing training efficiency with hardware memory constraints. Training was conducted for a maximum of 20 epochs with early stopping based on validation loss; in practice, convergence was achieved within 10–14 epochs. Attention masks are applied throughout to exclude padding tokens from the loss computation.

All experiments are implemented in PyTorch (version 2.10.0), utilizing the timm library for the Swin Transformer encoder and the Hugging Face Transformers library for ClinicalBERT and GPT-2. Training and evaluation are performed on an NVIDIA L4 GPU within Google Colab. During inference, reports are generated using beam search (beam width = 5), selected over greedy decoding for its ability to produce more globally coherent outputs. A repetition penalty of 1.5, a no-repeat trigram constraint, and a length penalty of 2.0 are applied to promote fluency, lexical diversity, and appropriate report length. The decoder was configured with a maximum sequence length of 512 tokens and a minimum length constraint to ensure sufficient clinical detail in all generated outputs.

The mask-guided attention mechanism within the visual encoder requires a lesion segmentation mask as an auxiliary input at both training and inference time. In the present study, the expert-annotated masks provided with the BrEaST dataset [11] are used throughout, enabling controlled evaluation of the proposed architecture under idealized segmentation conditions and isolating the contribution of the reporting framework from that of upstream segmentation error. This design does not, however, presuppose the continued availability of manual annotations in a deployed clinical setting.

In a prospective deployment scenario, the required masks would be produced automatically by an upstream segmentation module operating as a pre-processing stage, rather than being delineated manually by a radiologist. Contemporary breast ultrasound segmentation methods have demonstrated strong performance on this task; Himel and Chowdhury [17], for instance, report an Intersection over Union of 70.82% and a Dice coefficient of 80.56% on the BUSI and Thammasat benchmarks. Integrating such a network as a preceding stage would render the pipeline fully automated, requiring only the raw ultrasound image and structured clinical attributes as external inputs. Under this configuration, mask-guided attention would operate on predicted rather than ground-truth masks, and a degradation in report quality proportional to segmentation error would be anticipated.

The decoupling of segmentation and report generation into distinct, independently replaceable stages is a deliberate architectural choice: as breast ultrasound segmentation methods improve, the segmentation front-end may be upgraded without modification to the multimodal reporting components. A systematic evaluation of end-to-end performance using automatically generated masks, including quantification of the sensitivity of report quality to segmentation accuracy, is identified as an important direction for future work.

**Figure 2:**
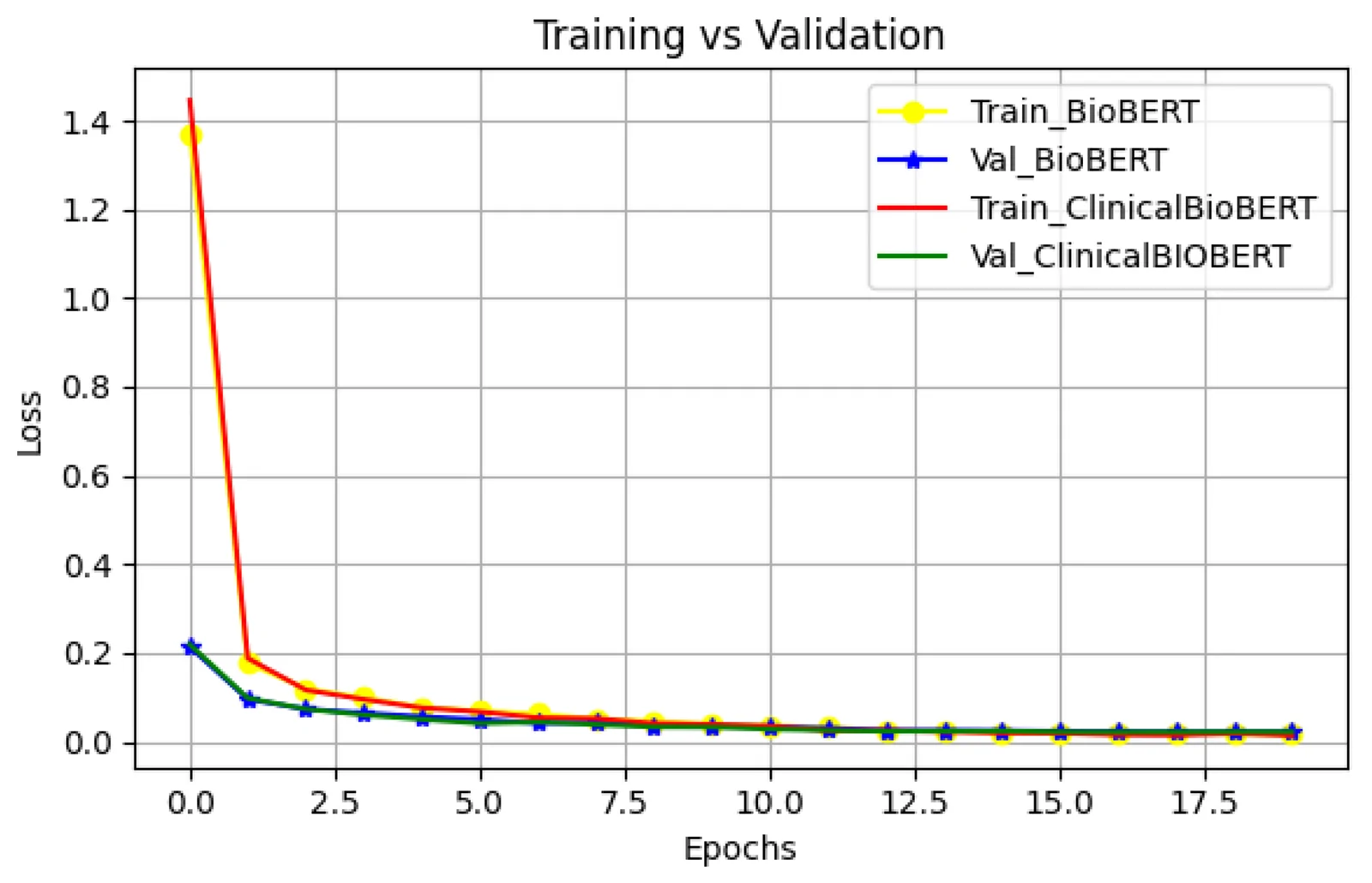
Training Loss vs. Validation Loss.

The training and validation loss curves in Figure 2 shows for both configurations on a shared axis, enabling direct comparison of generalization behaviour. In both models, the validation loss closely tracks the corresponding training loss throughout training, with no significant divergence observed between the two curves at any epoch. This alignment confirms the absence of overfitting, which is a noteworthy outcome given the relatively modest scale of the BrEaST dataset (256 cases). The use of a lightweight Swin-Small encoder, a dropout rate of 0.1 within the Transformer layers, and early stopping based on validation loss collectively contribute to this stable generalization behaviour.

A minor but consistent observation is that ClinicalBERT exhibits a slightly higher initial training loss (approximately 1.45) compared to BioBERT (approximately 1.35–1.40), which may reflect a marginally different parameter initialization or tokenization distribution at the outset of training. However, both models converge to comparable loss values by epoch 3–4, and this early difference has no discernible effect on final model performance. Both configurations typically reached convergence within 10–14 epochs, consistent with the early stopping criterion applied during training.

Taken together, the loss curves confirm that both model configurations train stably and generalize well under the given experimental setup. The near-identical convergence behaviour reinforces that the quantitative and qualitative performance gains observed with ClinicalBERT over BioBERT across BLEU, ROUGE-L, METEOR, and CIDEr, are attributable to the superior clinical language understanding conferred by ClinicalBERT domain-specific pre-training on real-world clinical notes, rather than to any difference in training dynamics or optimization efficiency.

### 5.1. Quantitative Analysis

To rigorously and systematically assess the performance of the proposed multimodal report generation framework, multiple experiments are conducted in which multiple encoder–decoder architecture combinations are explored and evaluated against one another. This comparative investigation was undertaken with the deliberate intention of isolating the contribution of individual architectural choices, particularly with respect to the selection of the textual encoder and the generative decoder, to the overall quality and coherence of the reports produced. The results obtained from these experiments, presented in full detail in Table 2, demonstrate clearly that both architectural decisions exert a meaningful and measurable influence on the linguistic fluency, clinical accuracy, and structural coherence of automatically generated radiological reports, underscoring the importance of principled component selection in the design of multimodal medical report generation systems as compared to our previous findings published recently using the same benchmark dataset [6].

Table 2 presents the quantitative performance of different encoder–decoder configurations for multimodal radiological report generation. The comparison focuses on two models using the same Swin-Small visual encoder and GPT-2 decoder, while varying the textual encoder between BioBERT and ClinicalBERT. The results clearly demonstrate that replacing BioBERT with ClinicalBERT leads to consistent improvements across all evaluation metrics. In particular, the ClinicalBERT-based model achieves a BLEU-4 score of 0.7252, compared to 0.6898 obtained with BioBERT. This improvement indicates better structural accuracy and more precise n-gram alignment with the reference reports.

Similarly, the ROUGE-L score increases from 0.7715 to 0.7900, reflecting improved sequence-level similarity and better preservation of report structure. The METEOR score also improves from 0.7888 to 0.8011, suggesting enhanced semantic alignment and more meaningful text generation. A notable improvement is observed in the CIDEr score, which increases from 1.7654 to 1.8897. Since CIDEr emphasizes the importance of domain-specific terminology, this result indicates that ClinicalBERT is more effective in generating clinically relevant and contextually appropriate descriptions. Overall, the results indicate that ClinicalBERT provides a more suitable textual representation for radiological report generation tasks. Its training on clinical data enables improved reasoning and interpretation, leading to more accurate and coherent reports. The consistent improvements across all metrics confirm the effectiveness of integrating ClinicalBERT within the proposed multimodal architecture.

### 5.2. Ablation Study

To isolate the contribution of each architectural component and verify that the observed performance is not attributable to any single element in isolation, a component-level ablation study was conducted. Five variants were derived from the full model, each differing by exactly one component: removal of mask-guided attention; replacement of the dual cross-attention fusion module with simple concatenation and linear projection; removal of the BI-RADS category from the structured text input; and two single-modality configurations in which either the text or the visual branch was removed entirely. All variants share the identical Swin-Small, ClinicalBERT, and GPT-2 backbone, are trained under the same protocol on the stratified partition described in Section 4.2 and are evaluated on the same held-out test set, ensuring that performance differences are attributable to the ablated component alone as reported in Table 3. All variants share the identical Swin-Small + ClinicalBERT + GPT-2 backbone, are trained on the stratified partition (*random_state =* 42), and evaluated on the held-out test set (*n =* 38). Each variant differs from the full model by exactly one component. Best result per metric in bold.

**Contribution of mask-guided attention:** Removing the mask-guided attention mechanism produces a substantial degradation across all metrics, with BLEU-4 declining from 0.7252 to 0.6231 (−14.1%) and CIDEr from 1.8897 to 1.1937 (−36.8%). The disproportionate magnitude of the CIDEr reduction relative to the BLEU-4 penalty indicates that the deterioration extends beyond surface phrasing to clinically distinctive content: without explicit lesion localizations, the visual encoder distributes representational capacity across the entire image field, including diagnostically uninformative background tissue and speckle artefacts. This finding empirically substantiates the design rationale that, given the low contrast and indistinct lesion boundaries characteristic of breast ultrasound, explicit spatial guidance is necessary for the visual encoder to extract lesion-specific features of sufficient quality to support clinically grounded generation.

**Contribution of dual cross-attention fusion:** Replacing the bidirectional cross-attention module with a concatenation-based fusion strategy produces the largest degradation observed among the component ablations on the two most semantically sensitive metrics: CIDEr falls to 1.1112 (−41.2%) and METEOR to 0.6010 (−25.0%), exceeding even the reduction caused by removing mask-guided attention. This is notable given that the BLEU-4 penalty (−13.8%) is marginally smaller than that of the mask ablation, indicating that the two components contribute through distinct mechanisms. As CIDEr applies TF-IDF weighting that privileges clinically distinctive terminology and METEOR incorporate synonym-level matching, the pattern demonstrates that the fusion mechanism contributes principally to semantic and clinical precision rather than to surface fluency. Concatenation permits the decoder to access both modalities but affords no mechanism by which visual and textual representations may condition one another prior to decoding; the resulting representation is therefore multimodal in composition but not in interaction.

**Contribution of the BI-RADS input attribute:** Excluding the BI-RADS category from the structured text input produces the smallest impact among the component ablations on surface-oriented measures (BLEU-4 −5.7%; ROUGE-L −0.9%), yet a considerably larger reduction in CIDEr (1.3957, −26.1%). This asymmetry is interpretable: the BI-RADS category constitutes a single discrete assessment within a report otherwise composed of morphological descriptors, and its omission therefore leaves the overall structure and phrasing of the generated text largely intact while removing a specific and clinically decisive propositional element. The magnitude of the CIDEr reduction confirms that the model does not reliably infer the assessment category from the remaining attributes and image evidence alone, indicating that BI-RADS supplies non-redundant diagnostic information.

**Modality contributions:** The two single-modality configurations reveal a pronounced asymmetry in modality dependence. The image-only variant degrades severely, with CIDEr falling from 1.8897 to 0.8660—a reduction of 54.2%—and BLEU-4 declining by 28.5% to 0.5182, the lowest value recorded across all configurations. Visual features alone are therefore insufficient to generate the clinically specific terminology required of a diagnostic report. Conversely, the text-only variant remains competitive on surface metrics (BLEU-4 0.7174, −1.1%) and marginally exceeds the full model on ROUGE-L (0.7950 versus 0.7900, +0.6%), reflecting that the structured clinical attributes supply much of the propositional content and sentence structure of the target report.

This asymmetry warrants careful interpretation rather than being construed as evidence that the visual branch is dispensable. ROUGE-L measures the longest common subsequence between generated and reference text and is therefore sensitive to structural conformity rather than diagnostic accuracy. The text-only configuration, conditioned exclusively on structured attributes, reproduces the templated report structure with high fidelity and consequently attains a marginally higher ROUGE-L score. Introducing visual conditioning permits limited departure from this template, incurring a negligible structural penalty while yielding a substantial gain in clinically distinctive content: CIDEr increases by 13.7% (1.6618 to 1.8897) and METEOR by 7.0% (0.7486 to 0.8011) when the visual branch is restored. The visual modality therefore contributes to diagnostic specificity and semantic grounding rather than to surface conformity—a trade-off that structural overlap metrics are inherently poorly positioned to capture, and one that favours clinical utility over template reproduction. This interpretation is corroborated by the explainability analysis presented in Section 5.4, in which Grad-CAM++ activations concentrate on the annotated lesion region and individual cross-attention heads exhibit functional specialization toward distinct clinical descriptors, demonstrating that visual evidence is actively engaged during generation.

The full model achieves the best performance on six of the seven evaluation metrics, and every ablated variant exhibits measurable degradation on the metrics most indicative of clinical quality. Dual cross-attention fusion and mask-guided attention emerge as the two most consequential components, accounting respectively for 41.2% and 36.8% of CIDEr performance, while the BI-RADS attribute and the visual modality contribute more selectively to diagnostic specificity. The sole exception—a marginal ROUGE-L advantage for the text-only configuration—reflects the structural bias of that metric rather than a deficiency in the multimodal design, as the same configuration is materially inferior on CIDEr and METEOR. These results collectively establish that the proposed architectural choices are individually justified rather than cumulatively assumed.

### 5.3. Qualitative Analysis

The qualitative comparisons between the ground truth reports and the outputs generated by different model configurations, including Swin (Small) + BioBERT and Swin (Small) + ClinicalBERT is shown in Table 4.

In the first example, the ground truth describes a lesion with oval shape, circumscribed margins, and hypoechoic characteristics, classified as BI-RADS 3 with a diagnosis of complex cyst. The BioBERT-based model partially captures the diagnostic conclusion but fails to generate a complete and coherent description of the lesion characteristics. In contrast, the ClinicalBERT-based model produces a more structured and consistent report, correctly identifying key features such as shape, margin, and echogenicity. However, a slight variation is observed in the interpretation, where the diagnosis is described as “cyst filled with thick fluid,” which is clinically related but not identical to the ground truth.

In the second example, both models correctly identify the lesion as irregular with indistinct margins and hypoechoic echogenicity. However, the BioBERT-based model underestimates severity by assigning a BI-RADS 4b classification, whereas the ClinicalBERT-based model accurately predicts BI-RADS 4c, matching the ground truth. This demonstrates the improved clinical reasoning capability of the ClinicalBERT-based model in assessing malignancy risk.

In the third example, the ground truth indicates a BI-RADS 5 classification with suspicion of malignancy. The BioBERT-based model introduces inconsistencies by incorrectly predicting tissue composition and assigning a lower BI-RADS category (4b). In contrast, the ClinicalBERT-based model produces a more accurate prediction, correctly identifying the BI-RADS 5 category and maintaining consistency in lesion description, indicating better alignment with clinical assessment.

In the fourth example, both models correctly capture the lesion shape and margin characteristics. However, the BioBERT-based model deviates in interpretation by predicting a complex cyst, while the ground truth indicates dysplasia. The ClinicalBERT-based model successfully aligns with the ground truth interpretation, demonstrating improved capability in generating clinically relevant conclusions.

Overall, the qualitative results highlight that while both models can generate coherent radiological descriptions, the ClinicalBERT-based model consistently produces more accurate and clinically meaningful reports. It shows better alignment with ground truth in terms of BI-RADS classification and interpretation, which are critical for diagnostic decision-making. These findings reinforce the quantitative results and confirm the effectiveness of ClinicalBERT in enhancing multimodal report generation.

### 5.4. Explainable AI (XAI) Analysis

To enhance the transparency and clinical reliability of the proposed multimodal report generation framework, multiple explainability techniques are employed to analyze the decision-making process of the model. Given the critical nature of medical applications, it is essential not only to achieve high performance but also to provide interpretable insights into how predictions are generated.

In this work, three prominent explainability methods are explored Grad-CAM, LIME, and SHAP. These techniques are selected to provide complementary perspectives, including spatial localization, region-based importance, and feature attribution. However, due to the multimodal and generative nature of the proposed architecture, not all methods are equally effective.

#### 5.4.1. Grad-CAM-Based Localization

**Figure 3:**
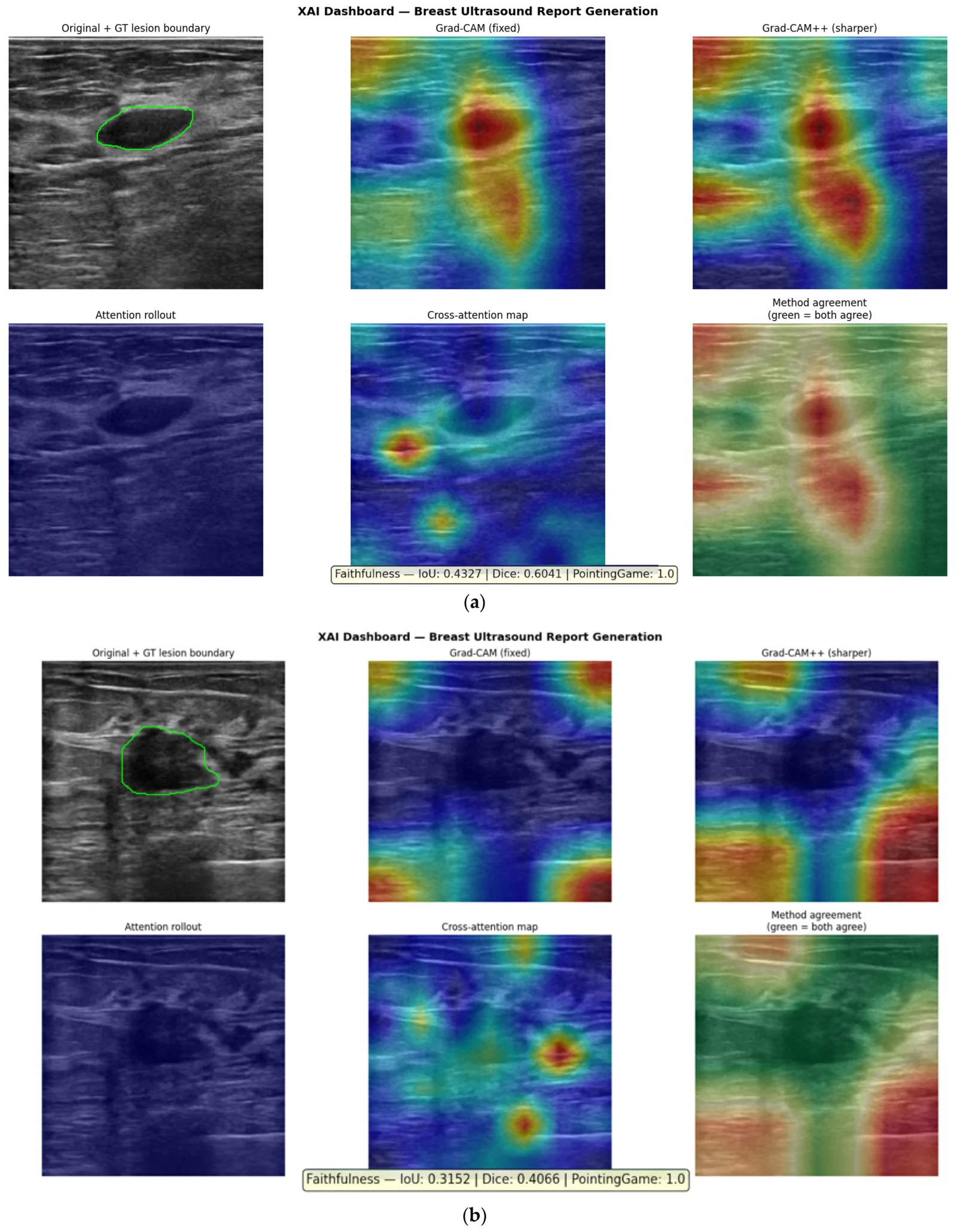
(**a**) XAI Dashboard from the BrEaST test set [14]—Example 1. (**b**) XAI Dashboard from the BrEaST test set [14]—Example 2.

The comprehensive explainability dashboard as shown in Figure 3a,b generated for a representative breast ultrasound case, consolidating the outputs of five complementary interpretability analyses alongside the original annotated image. The ground truth lesion boundary, delineated in green by an expert radiologist and visible in the top-left panel, provides a spatial reference against which the activation patterns produced by each explainability method can be evaluated for clinical correspondence and localization fidelity.

- **Grad-CAM and Grad-CAM++ Visualization**: The Grad-CAM activation map exhibits a pronounced concentration of high-importance activations centred directly upon the hypoechoic lesion region, with peak activations aligning closely with the lesion core as defined by the ground truth boundary. A moderate spread of intermediate activations extends into the immediately surrounding tissue, consistent with the clinical necessity of encoding margin characteristics relative to adjacent parenchymal structures. The Grad-CAM++ map produces a spatially tighter and more precisely localized activation pattern, attributable to its second-order gradient weighting formulation. The sharper activation boundary and reduced spread into non-lesion tissue confirm that Grad-CAM++ more faithfully isolates the regions assigned the highest discriminative weight by the model a particularly valuable property in breast ultrasound, where lesion boundaries are often ill-defined and spatially proximate to surrounding parenchyma.
- **Attention Rollout:** The attention rollout map displays a broadly diffuse, low-magnitude activation pattern distributed across the full spatial extent of the image. This result reflects the progressive spatial mixing that occurs as attention is accumulated across all four stages of the Swin-Small encoder hierarchy, wherein lesion-specific signals are increasingly diffused through successive window-based attention operations. This behaviour is consistent with observations in the transformer interpretability literature, where attention rollout characteristically produces more spatially uniform distributions than gradient-based methods due to cross-layer accumulation of attention weights encoding diverse spatial relationships.
- **Cross-Attention Visualization:** The cross-attention map reveals multiple discrete high-intensity regions distributed across the image, with primary activations at the lesion site and secondary activations in the inferior image region. In contrast to the singular peak observed in gradient-based maps, the multi-focal activation pattern reflects the fundamentally different information captured by cross-modal attention: each focus corresponds to the spatial alignment between specific clinical text tokens encompassing lesion shape, margin, echogenicity, and posterior acoustic descriptors and their most relevant visual counterparts. The inferior secondary activations are spatially consistent with posterior acoustic features in breast ultrasound, indicating that text tokens corresponding to posterior acoustic BI-RADS descriptors attend appropriately to the tissue region below the primary lesion.

- **Method Agreement:** The method agreement map illustrates spatial concordance between Grad-CAM++ and attention rollout activations. Strong agreement across background regions confirms that both methods consistently assign low importance to non-lesion tissue. Divergence at the lesion site where Grad-CAM++ produces a sharply localized peak and attention rollout yields a diffuse response reflects a methodological distinction rather than model inconsistency: the two methods capture complementary aspects of model behaviour, with gradient-based saliency highlighting discriminative importance at the final encoder layer and attention rollout characterizing cumulative spatial information flow across the full encoder hierarchy.
- **Faithfulness Evaluation:** Quantitative faithfulness metrics against the expert-annotated ground truth lesion boundary are also reported in Figure 3a,b. A Pointing Game accuracy of 1.0 confirms that the peak Grad-CAM++ activation falls precisely within the ground truth lesion region, demonstrating that the model’s most confident predictive evidence originates from the clinically correct anatomical location. A Dice coefficient of 0.6041 indicates moderately strong spatial overlap between the binarized activation map and the lesion mask, whilst an IoU of 0.4327 reflects the additional penalty for activation spread beyond the ground truth boundary. Collectively, these results provide empirical support for the clinical interpretability of the proposed model, confirming that its visual reasoning is grounded in diagnostically relevant lesion regions rather than spurious image features.

#### 5.4.2. Per-Head Cross-Attention Analysis

**Figure 4:**
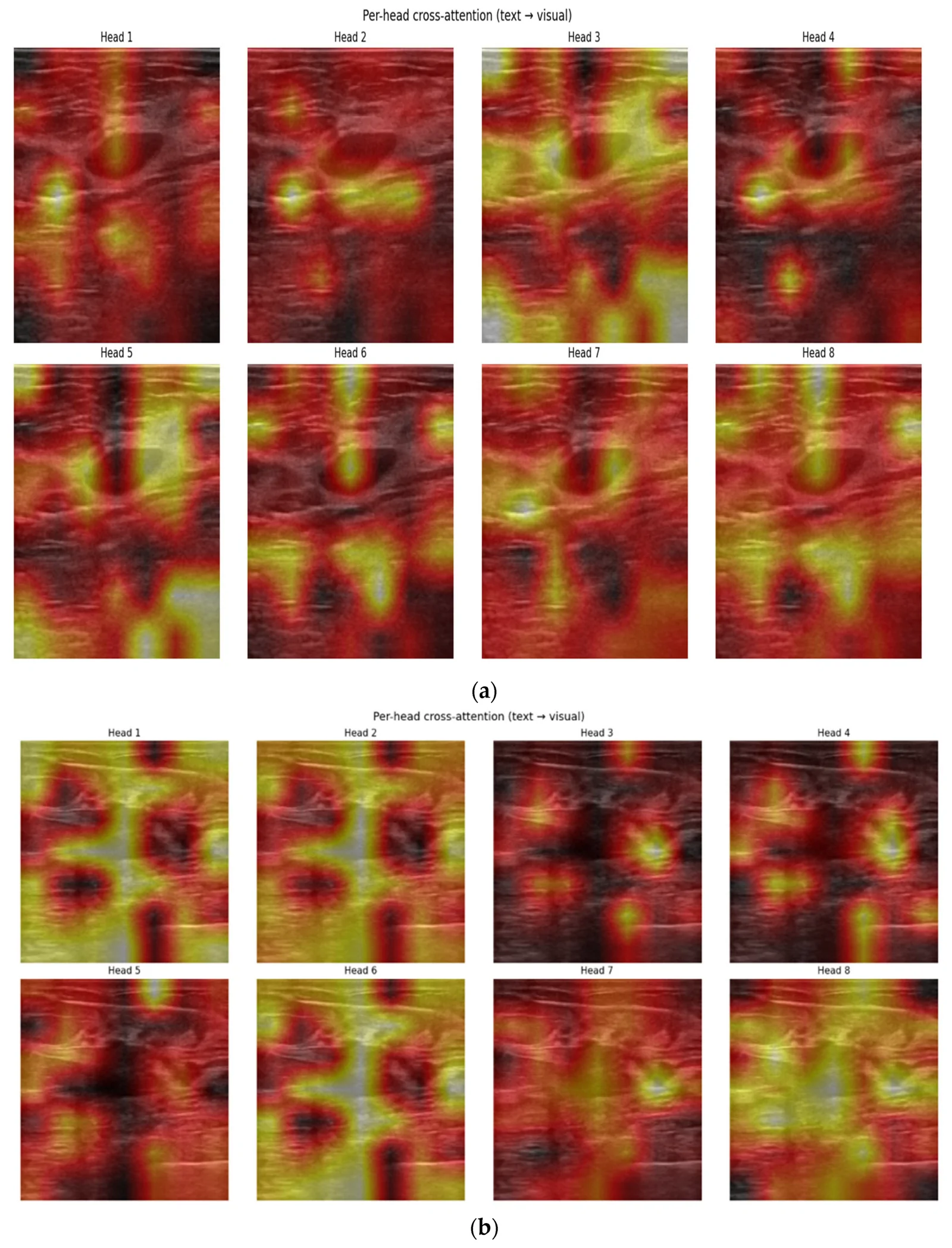
(**a**) Per-Head cross-attention (text-visual) from the BrEaST test set [11]—Example 1. (**b**) Per-Head cross-attention (text-visual) from the BrEaST test set [14]—Example 2.

In Figure 4a,b presents the per-head cross-attention maps generated by the dual cross-attention module, illustrating how text tokens distribute attention across visual feature representations across all eight heads. The diversity of activation patterns provides evidence that the multi-head mechanism has learned functionally differentiated spatial representations rather than converging on a uniform response—a characteristic indicative of well-regularized attention learning.

Heads 1, 2, and 4 exhibit the most clinically salient activations, producing concentrated high-intensity responses centred on the hypoechoic lesion region. Head 2 demonstrates the tightest localization, with a bright activation precisely overlapping the lesion body, suggesting a primary role in grounding shape and margin descriptors in image evidence. Head 1 produces a dual activation spanning the lesion centre and inferior boundary, consistent with sensitivity to lesion extent and boundary definition, whilst Head 4 exhibits broader morphological coverage likely encoding overall lesion geometry and spatial context.

Head 6 presents a notable activation in the tissue region immediately inferior to the lesion spatially consistent with posterior acoustic features in breast ultrasound, including posterior acoustic shadowing and enhancement, both of which are explicit BI-RADS descriptors within the structured clinical inputs. The emergence of a dedicated attention head sensitive to this region without explicit supervisory direction suggests that the model has implicitly learned the spatial association between posterior acoustic descriptors and their anatomical locus.

Head 7 displays a broadly diffuse activation across the full image extent with no pronounced lesion localization, consistent with encoding tissue-level attributes such as echogenicity and parenchymal composition inherently distributed properties necessary for BI-RADS category assessment. Heads 3 and 5 exhibit boundary-sensitive and laterally asymmetric patterns respectively: Head 3 produces bilateral suppression flanking the lesion whilst Head 5 responds prominently along the left lesion margin, suggesting complementary roles in margin characterization. Head 8 demonstrates an off-lesion activation in the upper image region, likely encoding background tissue reflectivity to support relative echogenicity representation.

Collectively, the observed functional differentiation across all eight heads confirms that the proposed dual cross-attention mechanism successfully decomposes the clinical visual interpretation task into specialized representational sub-problems encompassing lesion localization, boundary encoding, posterior acoustic detection, global tissue context, and echogenicity anchoring each contributing a distinct and complementary dimension of clinical evidence to report generation.

#### 5.4.3. LIME Based Regional Explanation

To complement gradient-based localization, Local Interpretable Model-Agnostic Explanations (LIME) was applied to provide region-level interpretability. LIME operates by perturbing segments (superpixels) of the input image and observing the impact on model predictions, thereby identifying regions that contribute most to the output.

**Figure 5:**
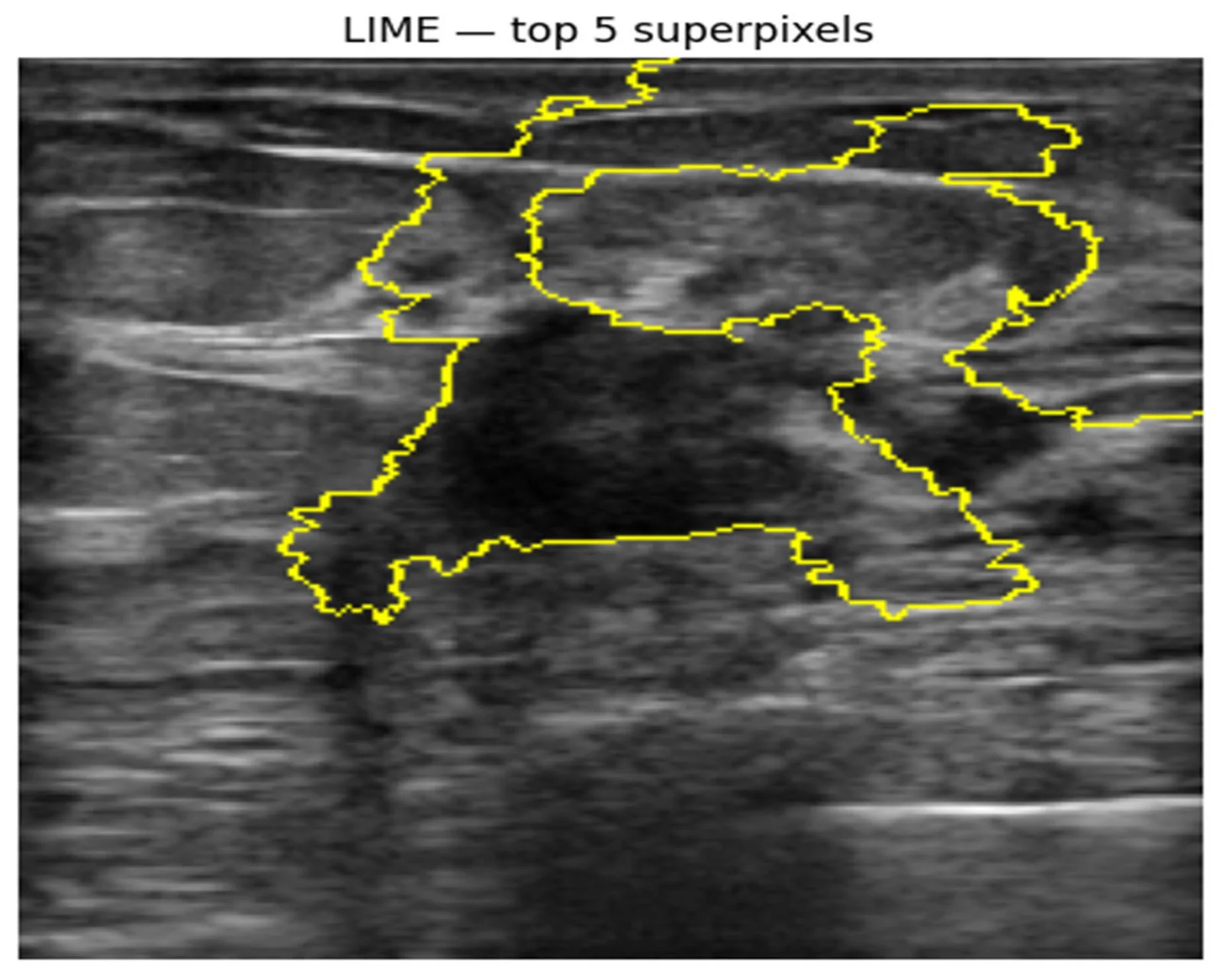
LIME Explanation from the BrEaST test set [14].

In this study, LIME was applied to ultrasound images using the LimeImageExplainer implementation with quick shift superpixel segmentation. For each explained case, 1000 perturbed samples were generated by randomly occluding subsets of superpixels, with occluding regions replaced by a zero-fill value. Because the proposed framework is generative rather than classificatory, the surrogate model was fitted against a continuous scalar score defined as the mean per-token log-likelihood of the reference report under the model, computed over non-padding tokens. This formulation quantifies how strongly each image region supports generation of the correct report, adapting LIME’s standard classification-oriented objective to the report generation setting. The five superpixels with the highest positive attribution weights are visualized as the regional explanation. Figure 5 shows the LIME results the primary lesion region was consistently identified as the most important contributor to the model’s prediction. Additional surrounding regions are also highlighted, indicating that the model incorporates contextual tissue information in its reasoning. Unlike Grad-CAM, which produces smooth heatmaps, LIME provides discrete region-based explanations. This difference highlights that the model relies not only on localized lesion features but also on broader contextual patterns present in the surrounding tissue.

#### 5.4.4. Token Level Explanation

**Figure 6:**
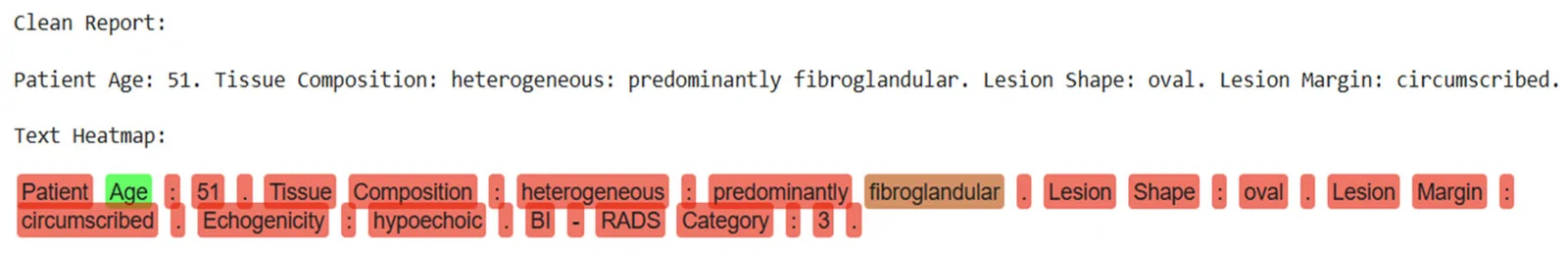
Text Heatmaps.

Token-level attention analysis shows that clinically significant terms such as “hypoechoic”, “oval”, and “circumscribed” receive higher importance scores, while non-diagnostic information receives lower attention as shown in Figure 6. This demonstrates that the model prioritizes meaningful medical features during report generation, ensuring both interpretability and clinical relevance.

The text heatmaps reveals that the model assigns higher importance to clinically relevant descriptors, such as “hypoechoic”, “oval”, and “circumscribed”. These terms are critical in breast ultrasound interpretation, as they relate to lesion characterization and diagnostic decision-making.

In contrast, non-diagnostic information such as “Patient Age” and numerical values exhibit relatively lower importance scores. This indicates that the model effectively prioritizes medically significant features over auxiliary or demographic information during report generation. Additionally, intermediate importance is observed for contextual descriptors such as “heterogeneous” and “fibroglandular”, suggesting that the model incorporates surrounding tissue characteristics as part of its reasoning process.

## 6. Discussion

The findings of this study demonstrate that the proposed explainable multimodal framework effectively integrates hierarchical visual feature extraction and biomedical language modelling to generate clinically meaningful breast ultrasound reports. The strong performance achieved across BLEU, ROUGE, METEOR, and CIDEr, indicates that the generated reports exhibit high linguistic quality while maintaining semantic consistency with expert-written reports. Beyond quantitative performance, the explainability analysis provides additional evidence that the model establishes meaningful associations between lesion characteristics and the generated clinical descriptions, thereby improving the interpretability of the reporting process. Compared with our previous study [6], which demonstrated the feasibility of applying vision-language transformers for breast ultrasound report generation, the proposed framework extends this approach by incorporating a Swin Transformer for hierarchical feature extraction, mask-guided attention to emphasize lesion regions, and a dual cross-attention mechanism for enhanced visual-textual fusion. These architectural improvements enable more effective modelling of lesion morphology and multimodal interactions, contributing to the improved semantic quality and clinical relevance of the generated reports while providing greater transparency through integrated explainability.

Despite these promising findings, several limitations should be acknowledged. The proposed framework was evaluated using a single publicly available breast ultrasound dataset, which may limit the generalizability of the results to more diverse clinical settings involving different patient populations, imaging protocols, and ultrasound equipment. In addition, the model relies solely on imaging data, whereas routine clinical reporting often incorporates complementary information such as patient history, BI-RADS assessment, pathology findings, and prior examinations. Furthermore, although automated evaluation metrics provide a comprehensive assessment of linguistic and semantic quality, they cannot fully measure clinical correctness or diagnostic utility. Future work should therefore focus on validating the framework using large multi-centre datasets, incorporating additional clinical information into the multimodal learning process, and conducting expert evaluations with radiologists to assess the clinical accuracy, reliability, and practical applicability of the generated reports in real-world healthcare environments.

## 7. Conclusions

This study proposed an explainable multimodal framework for automated breast ultrasound report generation by integrating a Swin Transformer visual encoder, a BioBERT text encoder, a GPT-2 language decoder, and a dual cross-attention mechanism. The framework was designed to improve lesion representation, strengthen visual-textual alignment, and generate clinically meaningful radiology reports while providing transparent explanations for the model’s predictions.

The experimental results demonstrate that the proposed framework effectively captures lesion-level characteristics through the combination of Swin Transformer feature extraction and mask-guided attention. Grad-CAM visualizations confirmed that the model consistently focused on clinically relevant lesion regions rather than surrounding background tissue, indicating that the generated diagnostic descriptions were based on meaningful image evidence. Furthermore, the dual cross-attention mechanism successfully aligned visual features with textual representations, as demonstrated by the strong correspondence between lesion activation maps and token-level importance scores. This alignment contributed to the generation of coherent, clinically relevant, and semantically accurate reports.

The quantitative evaluation further validates the effectiveness of the proposed framework. Strong performance across BLEU, ROUGE, METEOR, and CIDEr demonstrates the model’s ability to generate fluent, contextually appropriate, and clinically consistent reports that closely resemble expert-written radiology descriptions. The complementary use of traditional natural language generation metrics together with semantic similarity measures provides evidence that the proposed approach achieves both high linguistic quality and meaningful clinical interpretation.

The integration of explainable AI techniques represents another important contribution of this work. By combining Grad-CAM visual explanations with token-level attention analysis, the proposed framework provides interpretable evidence supporting its diagnostic predictions. This level of transparency improves the trustworthiness of the generated reports and offers clinicians greater confidence in understanding how specific image regions influence the generated clinical descriptions.

Although this study did not include a direct experimental comparison with CNN-based report generation models, the obtained results, together with findings reported in the existing literature, indicate that transformer-based architectures equipped with domain-specific language models such as BioBERT provide superior semantic representation, contextual understanding, and multimodal feature fusion for medical report generation. These capabilities enable the production of reports that are more clinically coherent and better aligned with expert annotations.

Future work will focus on evaluating the model on larger multi-centre breast ultrasound datasets with greater diversity in patient populations and imaging conditions. Additional research will investigate external clinical validation, integration of advanced large vision-language models, incorporation of multimodal clinical information such as patient history and radiologist notes, and prospective evaluation in real clinical workflows. These extensions will further improve the robustness, generalizability, and clinical applicability of explainable AI systems for automated breast ultrasound diagnosis and report generation.

## Figures and Tables

**Table 1:** Class distribution across the training, validation, and test partitions.

Subset	Total	Benign	Malignant
Training (70%)	176	108	68
Validation (15%)	38	23	15
Test (15%)	38	23	15
Total	252	154	98

**Table 2:** Evaluation and comparison with (Khatoon & Mahmood, 2026) [6] of different encoder–decoder architecture combinations for generating Breast Ultrasound Diagnostics.

Model Configuration	BLEU-1	BLEU-2	BLEU-3	BLEU-4	ROUGH-L	METEOR	CIDEr
ViT + BERT + TransformerDecoder [6]	0.590	0.435	0.305	0.135	0.590	0.360	0.880
ViT + BioBERT + TransformerDecoder [6]	0.635	0.495	0.345	0.160	0.614	0.395	1.00
ViT + BioBERT as Encoder + Decoder [6]	0.665	0.525	0.375	0.175	0.648	0.425	1.08
ViT + BioBERTEncoder + GPT2Decoder [6]	0.650	0.510	0.360	0.180	0.670	0.440	1.05
ViT + GPT2asEncoder + Decoder [6]	0.555	0.415	0.285	0.160	0.650	0.435	0.820
**This Study**—Swin (Small) + BioBERT Encoder + GPT2 Decoder	0.7299	0.7159	0.7052	0.6898	0.7715	0.7888	1.7654
**This Study**—Swin (Small) + Clinical BioBERT + GPT2 Decoder	**0.7602**	**0.7490**	**0.7396**	**0.7252**	**0.7900**	**0.8011**	**1.8897**

**Table 3:** Component-level ablation study.

Configuration	BLUE-1	BLUE-2	BLUE-3	BLUE-4	ROUGE-L	METEOR	CIDEr
Full Model	**0.7602**	**0.7490**	**0.7396**	**0.7252**	**0.7900**	**0.8011**	**1.8897**
Without Mak-Guided Attention	0.6337	0.6299	0.6271	0.6231	0.6950	0.6503	1.1937
Without Dual Cross-Attention	0.6627	0.6458	0.6306	0.6249	0.6966	0.6010	1.1112
Without BI-RADS input	0.7171	0.7066	0.6962	0.6839	0.7828	0.7363	1.3957
Image-only	0.6382	0.5984	0.5612	0.5182	0.7011	0.6522	0.8660
Text-only	0.7264	0.7235	0.7209	0.7174	0.7950	0.7486	1.6618

**Table 4:** Performance Comparison of Multimodal Encoder–Decoder Architectures for Generating Breast Ultrasound Diagnostics.

Images	Ground Truth	Swin (Small) + BioBERT Encoder + GPT2 Decoder	Swin (Small) + Clinical BioBERT + GPT2 Decoder
* 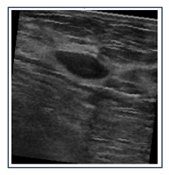 *	The breast tissue is heterogeneous: predominantly fibroglandular. The lesion appears oval in shape with circumscribed margins. It is hypoechoic in echogenicity. Based on these findings, it is classified as BI-RADS 3. The findings are suggestive of Complex cyst/Non-simple cyst.	e.g., microlobulated breast tissue. Based on these findings, it is classified as BI-RADS 3. The findings are suggestive of Complex cyst/Non-simple cyst.	The lesion appears oval in shape with circumscribed margins. It is hypoechoic in echogenicity. Based on these findings, it is classified as BI-RADS 3. The findings are suggestive of Cyst filled with thick fluid.
* 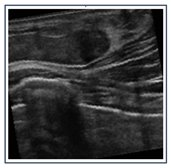 *	The breast tissue is homogeneous: fibroglandular. The lesion appears irregular in shape with not circumscribed—indistinct margins. It is hypoechoic in echogenicity. Based on these findings, it is classified as BI-RADS 4c. The findings are suggestive of Suspicion of malignancy.	The lesion appears irregular in shape with not circumscribed—indistinct margins. It is hypoechoic in echogenicity. Based on these findings, it is classified as BI-RADS 4b. The findings are suggestive of Suspicion of malignancy.	The lesion appears irregular in shape with not circumscribed—indistinct margins. It is hypoechoic in echogenicity. Based on these findings, it is classified as BI-RADS 4c. The findings are suggestive of Suspicion of malignancy.
* 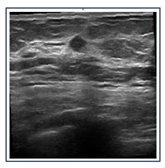 *	The breast tissue is not available. The lesion appears irregular in shape with not circumscribed—indistinct margins. It is hypoechoic in echogenicity. Based on these findings, it is classified as BI-RADS 5. The findings are suggestive of Suspicion of malignancy.	The breast tissue is homogeneous: fat. The lesion appears irregular in shape with not circumscribed—indistinct margins. It is hypoechoic in echogenicity. Based on these findings, it is classified as BI-RADS 4b. The findings are suggestive of Suspicion of malignancy.	The lesion appears irregular in shape with not circumscribed—indistinct margins. It is hypoechoic in echogenicity. Based on these findings, it is classified as BI-RADS 5. The findings are suggestive of Suspicion of malignancy.
* 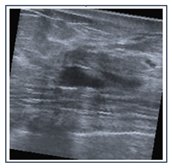 *	The breast tissue is heterogeneous: predominantly fibroglandular. The lesion appears oval in shape with circumscribed margins. It is complex cystic/solid in echogenicity. Based on these findings, it is classified as BI-RADS 4a. The findings are suggestive of Dysplasia.	The lesion appears oval in shape with circumscribed margins. It is heterogeneous in echogenicity. Based on these findings, it is classified as BI-RADS 4a. The findings are suggestive of Complex cyst/Non-simple cyst.	The lesion appears oval in shape with circumscribed margins. It is heterogeneous in echogenicity. Based on these findings, it is classified as BI-RADS 4a. The findings are suggestive of Dysplasia.

## Data Availability

The original data presented in the study are openly available in the “Cancer Imaging Archive (TCIA)” in the Breast Lesions Ultrasound (USG) collection available at: https://www.cancerimagingarchive.net/collection/breast-lesions-usg/ (accessed on 10 March 2026).
